# Decreasing relatedness among mycorrhizal fungi in a shared plant network increases fungal network size but not plant benefit

**DOI:** 10.1111/ele.13947

**Published:** 2021-12-31

**Authors:** Anouk van 't Padje, Malin Klein, Victor Caldas, Loreto Oyarte Galvez, Cathleen Broersma, Nicky Hoebe, Ian R. Sanders, Thomas Shimizu, E. Toby Kiers

**Affiliations:** ^1^ Laboratory of Genetics Wageningen University & Research Wageningen the Netherlands; ^2^ Department of Ecological Sciences Faculty of Earth and Life Sciences Vrije Universiteit Amsterdam Amsterdam the Netherlands; ^3^ AMOLF Institute Amsterdam the Netherlands; ^4^ Departent of Ecology and Evolution University of Lausanne Lausanne Switzerland

**Keywords:** arbuscular mycorrhizal fungi, conflict, cooperation, genetic relatedness, quantum‐dot

## Abstract

Theory suggests that relatives will cooperate more, and compete less, because of an increased benefit for shared genes. In symbiotic partnerships, hosts may benefit from interacting with highly related symbionts because there is less conflict among the symbionts. This has been difficult to test empirically. We used the arbuscular mycorrhizal symbiosis to study the effects of fungal relatedness on host and fungal benefits, creating fungal networks varying in relatedness between two hosts, both in soil and in‐vitro. To determine how fungal relatedness affected overall transfer of nutrients, we fluorescently tagged phosphorus and quantified resource distribution between two root systems. We found that colonization by less‐related fungi was associated with increased fungal growth, lower transport of nutrients across the network, and lower plant benefit ‐ likely an outcome of increased fungal competition. More generally, we demonstrate how symbiont relatedness can mediate benefits of symbioses.

## Graphical abstract

We used the arbuscular mycorrhizal symbiosis to study the effects of fungal relatedness on host and fungal benefits, creating fungal networks varying in relatedness between two hosts. To determine how fungal relatedness affected overall transfer of nutrients, we fluorescently tagged phosphorus and quantified resource distribution between two root systems. We found that decreased relatedness in a fungal network was associated with increased fungal growth, lower transport of nutrients across the network, and lower plant benefit ‐ likely an outcome of increased fungal competition.

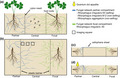

## INTRODUCTION

A key prediction in evolutionary biology is that relatives will cooperate more and compete less (Griffin & West, 2002; West et al., 2007; West, Pen, et al., 2002). Kin selection emerges because relatives share high proportions of their genes, and by cooperating, more of these genes are passed on to the next generation. Kin selection has been applied to understand a diversity of cooperative phenomena, from cooperation among gut microbiota (Simonet & McNally, 2021) to cooperation among humans (Apicella & Silk, 2019). However, kin selection can also be vulnerable to competition among relatives, especially in cases where there is high spatial structuring in the population. Under these cases, increased competition among relatives can totally negate benefits of cooperation (Queller, 1992; West, Pen, et al., 2002).

Manipulating relative relatedness in symbiotic communities is challenging. This has complicated empirical tests on cooperation and conflict. Here, we used the arbuscular mycorrhizal symbiosis to study the effects of symbiont relatedness on their hosts which interact via competing or cooperating mycorrhizal fungal networks. The vast majority of land plants are colonised by arbuscular mycorrhizal fungi. The fungi exchange soil bound nutrients such as phosphorus and nitrogen for photosynthetic carbon from the host plant (Jiang et al., 2017; Keymer et al., 2017; Luginbuehl et al., 2017). The fungi form underground networks that can connect roots of different plants. Hyphal fusion, known as anastomosis, can occur among fungi of the same strain (Giovannetti et al., 2004; Jakobsen, 2004; Mikkelsen et al., 2008). This has the potential to increase resource sharing across the fungal network (Johansen & Jensen, 1996; Walder et al., 2012), which could increase the fitness of the fungi (Giovannetti et al., 2015) and potentially their hosts (Roger et al., 2013). In contrast, when fungi are genetically less related, the hyphae are vegetatively incompatible and fusion will not occur (Croll et al., 2009; Giovannetti et al., 2003). Direct antagonism among competing mycorrhizal strains has been shown to lead to negative outcomes for fungal abundance and plant growth (Engelmoer et al., 2014), and can also influence fungal co‐existence within host roots (Roger et al., 2013). Competition between distantly related fungal isolates resulted in almost complete exclusion of one isolate by the other, whereas more related isolates shared the roots space in an almost 50:50 proportion (Roger et al., 2013).

While these data suggest that level of relatedness can affect fungal competitive dynamics within a host, it is unknown how relatedness affects functioning of the hyphal network itself ‐ especially when the hyphae connect multiple plants. For example, hosts may benefit from interacting with highly related strains because of reduced conflict and enhanced competition within the community (Frank, 1996a, 2003; West, Pen, et al., 2002). However, interacting with less‐related strains may also be beneficial for hosts. Specifically, if there is a greater relative difference among the symbiont species in their ability to acquire different, or complementary resources, hosts could benefit from interacting with non‐relatives (Argüello et al., 2016; Jansa et al., 2008; Wagg et al., 2011).

Our aim was to understand how fungal relatedness affects the physical formation and nutrient transfer in a fungal network formed between hosts. To study phosphorus distribution and transfer, we employed a technique in which we tag phosphorus rock (apatite) with fluorescent quantum‐dot nanoparticles (van 't Padje, Oyarte Galvez, et al., 2020; van 't Padje, Werner 2020; van 't Padje et al., 2021; Whiteside et al., 2019) Quantum‐dots fluoresce in bright and pure colours when excited with UV‐light (Färkkilä et al., 2021). We used a class of quantum‐dots that were highly fluorescent, and stable (Gustafsson et al., 2015; Whiteside et al., 2009). Past work has shown that quantum‐dot‐apatite can be taken up by fungal hyphae (van 't Padje, Oyarte Galvez, et al. (2020), video S1), and while the precise mechanisms are unknown, uptake likely relies on dissolution, followed by endocytic processes, commonly observed in fungi (Alloush & Clark, 2001; Pel et al., 2018; Powell & Daniel, 1978). In addition, quantum‐dot‐apatite has been shown to accumulate in growing root and leaf tissue, as is expected with nutrients allocated to building new tissue (van 't Padje et al., 2021; Whiteside et al., 2019). While more work is needed in determining how florescence measurements relate to absolute values of phosphorus accumulation in host tissue (Färkkilä et al., 2021), the technique gives a useful proxy to compare relative rates of transfer from fungal networks to host roots across treatments (Supporting Information).

To study the effects of fungal relatedness on host and fungal benefits, we first grew a host root colonised by a single focal strain. The fungi of this focal plant were allowed to interact with a fungal network of a second host plant that was colonised by the same genotype (“selfing”, which allowed for fusion), or two genetically less‐related fungal strains (“non‐selfing”, in which there was no fusion). In order of highest to lowest relatedness, these treatments included: (i) the same fungal genotype (selfing), (ii) a different fungal strain within the same species (non‐selfing), or (iii) a fungal strain of a different species in the same genus (non‐selfing). We grew these plant and fungal treatments as both whole‐plants in soil and as in‐vitro root organ cultures in petri plates. The latter allowed us to determine where phosphorus was distributed across the network using our quantum‐dot tagging technique, as well as to describe the physical fungal network structure using imaging techniques (Boddy, 1999; Heaton, López, et al., 2012; Heaton, Obara, et al., 2012). In selfing networks, we assumed that resources are shared equally across networks of fungal strains because they are a single, selfing genotype. We then asked how selfing and non‐selfing fungal networks between the two hosts influenced: (i) host growth, (ii) fungal colonisation inside root tissue (intraradical colonisation), (iii) network formation outside the root tissue (extraradical colonisation), and (iv) transfer of nutrients across the network to the host root.

## MATERIALS AND METHODS

### Experimental design

**FIGURE 1:**
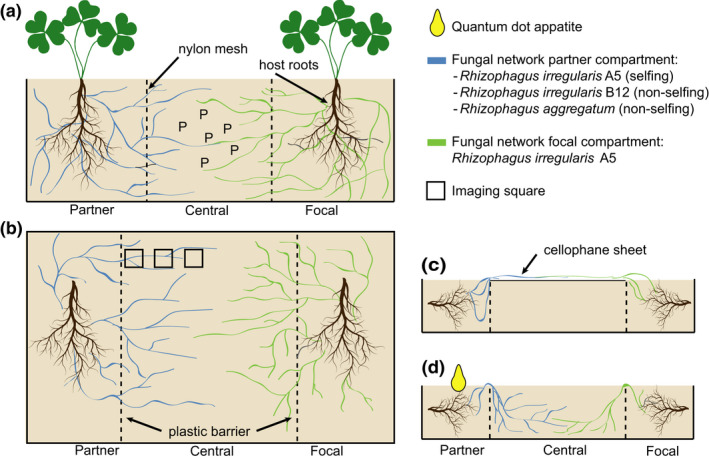
Experimental design. (a) We grew two host plants of *Medicago truncatula* in an elongated box divided into three compartments by nylon mesh. The focal plant grew in the focal compartment and was inoculated with *Rhizophagus irregularis* strain A5 (A5). The partner plant grew in the partner compartment and was inoculated with either A5, *R. irregularis* strain B12 (B12) or *R. aggregatum* (Agg). Only the central compartment was supplied with phosphorus (P) in the form of apatite. (b) We grew two host roots of in‐vitro *Daucus carota* on a rectangular plate. The focal host root grew in the focal compartment, and was inoculated with A5. The partner plant grew in the partner compartment and was inoculated with either A5, B12 or Agg. The fungal hyphae from both root systems could cross over the plastic barrier into the central compartment, but the plastic barrier prevented the diffusion of nutrients. We imaged the central compartment in three locations (black squares) to study fungal architecture. (c) Side view of an in‐vitro plate in which the central compartment was covered with a cellophane sheet to allow for 2D fungal imaging. (d) Side view of an in‐vitro plate without cellophane sheet. The fungal hyphae could cross over the plastic barrier into the medium of the central compartment. We then added quantum‐dot‐apatite to the partner compartment to quantify the transfer of quantum‐dot‐apatite from partner roots across the fungal network and into focal roots

In both whole‐plant greenhouse and in‐vitro root organ culture experiments, we employed a basic three‐compartment setup (Olsson et al., 2014). One compartment contained the focal plant or root, which was then consistently inoculated with the model strain *Rhizophagus irregularis* strain A5 (Sanders Lab, hereafter A5). The second compartment contained a second root inoculated with one of three fungal treatments, one selfing: *R. irregularis* A5, and two non‐selfing fungi: *R*. *irregularis* strain B12 (Sanders Lab, hereafter B12) or *R. aggregatum* (Agg), listed in order of decreasing relatedness to the focal strain (Roger et al., 2013). These strains were chosen because they allowed us to test three levels of relatedness in a genetically well‐characterised genus (Roger et al., 2013). In both the whole‐plant and in‐vitro setup, the roots compartments were physically separated by a 'fungus‐only' compartment in which the fungi from the two hosts could directly interact (Figure 1a,b). To study the physical structure of fungal networks in in‐vitro root organ cultures, we covered the fungus‐only compartment with a cellophane sheet to restrict network growth to 2D top layer (Crawford et al., 1993; Hitchcock et al., 1996; Ritz et al., 1996) (Figure 1c). To determine the nutrient transport from the fungal network into the host roots, we added quantum‐dot‐apatite to the partner compartment of the in‐vitro root organ cultures and determined how much was transferred to the focal root (Figure 1d).

### Whole‐plant greenhouse experiment

#### Germination and growing conditions

We first performed whole‐plant greenhouse experiments. We used *Medicago truncatula* as a host (genotype Jemalong A17, courtesy of dr. Bettina Hause, Leibniz Institute of Plant Biochemistry), as previously (Kiers et al., 2011; Werner et al., 2018; Whiteside et al., 2019). We scarified *M. truncatula* seeds by submerging them in 95% sulfuric acid for 5–10 min; subsequently, we rinsed the seeds with ddH_2_O. We stored the seeds in petri dishes on moist filters, first 2 days in the dark at 5°C, then one day at 20°C in the dark, followed by two days in the light at 20°C. We planted germinated seeds in autoclaved germination soil (RHP Agra‐vermiculite (M3)). After 11 days, we selected seedlings of 3–4 cm with at least three leaves to transplant to three‐compartment 6 L boxes (Garcia et al., 2006). The boxes were divided into three equal compartments with a 50 µm pore size nylon mesh (Cell Micro Sieves, Gentaur). This limited the plant roots to the outermost compartments, but allowed the fungal hyphae to grow in the central compartment. We filled each compartment with autoclaved quartz sand and supplemented the central compartment with 1 g hydroxyapatite per kilogram quartz sand as a phosphorus source (Pel et al., 2018). We planted one *M. truncatula* seedling in each of the two outer compartments of the three‐compartment box.

As fungal inoculum, we homogenised in‐vitro Ri T‐DNA *Daucus carota* L. transformed root organ cultures containing each fungus and added 16 ml of the mixture to the roots (~700 spores). We randomly assigned one plant as the “focal plant”. This focal plant was consistently inoculated with the strain 'A5'. The other root was designated as the partner plant, and inoculated with either A5 (*n *= 8), B12 (*n *= 9) or Agg (*n *= 9) (Figure 1a). After inoculation, we added 10 ml water to the roots and fertilised the plants once with an adjusted Hoagland solution containing 25% phosphorus of the original concentration (5.5 mM KNO_3_; 4.0 mM CaCl_2_·2H_2_O; 7.25 mM NH_4_NO_3_; 0.5 mM KH_2_PO_4_; 1.0 mM; 20 mM MgSO_4_·6H_2_O; Fe(Na)EDTA; 1.0 ml/L micronutrients). We placed all pots in a randomised grid, and rotated them every 2 weeks in the greenhouse, with temperatures between 20–30°C. After a week, we covered the sand with a one cm layer of sterile low‐density polyethylene beads (Fardem Packaging) to limit evaporation. We watered the plants twice a week with dH_2_O, keeping the water content between 10%–12.5% and fertilised the plants every 2 weeks with 35 ml of an adjusted Hoagland solution, containing no phosphorus but extra nitrogen (5.5 mM KNO_3_; 4.0 mM CaCl_2_·2H_2_O; 7.25 mM NH_4_NO_3_; 0.5 mM KCl; 1.0 mM MgSO_4_·6H_2_O; 20 mM Fe(Na)EDTA; 1.0 ml/L micronutrients). To confirm that our plants did not differ in plant biomass when grown with each of the three fungal strains, we also grew single *M*. *truncatula* plants in standard 880 ml pots, filled with sterile quartz sand. We inoculated host plants with either A5, B12, or Agg, and grew plants as above. We found no statistically significant difference in either root or shoot biomass of plants grown with our three strains. Although the pot size of the whole‐plant experiment and the control experiment differed, this confirmed that the fungal strains did not differ significantly in their nutrient provisioning (Figure S3).

#### Harvest

We harvested the plants after eight weeks and separated shoot from root just below the rosette formation. We stored shoots in paper bags and dried the material at 70°C. We washed sand from the roots, homogenised them, weighed root material, took subsamples for DNA isolation, stored subsamples at −20°C and dried the remaining roots material in paper bags at 70°C.

### In‐vitro root organ cultures

#### Inoculation and growing conditions

We then performed in‐vitro root organ culture experiments. To create a three‐compartment in vitro system, we modified a 4‐well compartment system by removing the central barrier, creating a large central fungal compartment and two smaller root compartments (Olsson et al., 2014). We filled each compartment with Modified Strullu Romand (MSR) media (0.4% phytagel, pH 5.5, 55 nM sucrose, 3980 µM N, 30 µM P, Fortin et al., 2002). To each focal and partner root compartment, we transplanted a branching, two cm long section of in‐vitro Ri T‐DNA *Daucus carota* L. transformed root organ culture. We inoculated the roots with a 1 × 1 cm^2^ agar plug containing ~700 fungal spores. We again randomly designated one root as the “focal root”, and inoculated it with *R. irregularis* A5. The partner root was inoculated with either A5, B12 or Agg (Figure 1b). In the A5‐A5 treatment, the two compartments were randomly assigned as focal or partner. We sealed the plates with parafilm and stored them in the dark at 25°C. We placed any roots crossing into the central compartment back into the root compartment using sterile equipment.

#### Image analysis

To image and quantify the growth of the extraradical fungal network, we covered the central compartment of a random subset of plates (A5: *n *= 12, B12: *n *= 12, Agg: *n *= 17) with sterile cellophane (Figure 1c). We monitored plates for fungal growth in the focal and partner compartment and checked weekly for fungal cross over into the central compartment. After approximately 20 days, the first hyphae crossed the plastic barrier to the central compartment. We then imaged the entire fungal network in the central compartment using a 5× objective on a Leica Wild M8 preparation microscope, taking images with an Olympus SC180 camera. To study network formation in the absence of the focal strain, we also grew each strain individually and imaged the fungal network. In this case, the images were obtained using a high‐resolution camera, 12.3 MP resolution (Basler acA4112‐30um), together with a long‐working distance objective (TL2X‐SAP ‐ 2X Super Apochromatic).

We selected three locations with a dimension of 5 × 5 mm^2^ (640 × 640 px^2^) across the central compartment in each of the treatments, as well as three locations across networks grown singly. The locations ranged across the space connecting the partner compartment barrier to the centre of the central compartment (Figure 1b). Using MATLAB, we applied morphological operations to the images, binarised the images, removed isolated cluster (background noise) and extracted the network skeleton of the extraradical fungi. We calculated the mass fractal dimension (*D_m_*) of every spatial area using the box‐counting technique (Boddy & Donnelly., 2008; Bouda et al., 2016; Falconer, 2003; Hitchcock et al., 1996), with a square grid size ranging from 8 to 64 pixels, i.e., from ^1^/_10_ to ^1^/_80_ times the total square area. We then estimated the fractal dimension by

$$N\left(s\right)\propto s^{‐\;D_{m}}$$

where *s* corresponds to the grid size and *N(s)* the total number of boxes that contain fungal hyphae. We calculated the density of the network (surface percentage) as the ratio between the surface occupied by the network and the total square area.

#### Nutrient transfer

To determine nutrient transport across the fungal network and into the root growing in the focal compartment, we used a second subset of the in‐vitro plates (A5: *n *= 12, B12: *n* = 8, Agg: *n* = 12) in which we injected quantum‐dot‐apatite as a fluorescently labelled phosphorus source in the partner root compartment. We constructed green (490 nm) quantum‐dot‐apatite by conjugating hydroxyapatite with fluorescent quantum‐dots following the technique described in Whiteside et al. (2019). We injected 500 µl of a 126 mM phosphorus solution to the partner compartment of each replicate (Figure 1d), and harvested these plates 2 weeks after quantum‐dot‐apatite injection. This allowed us to compare relative phosphorus transfer from one root compartment to another via the mycorrhizal network in each treatment.

#### Harvest, fluorescent analysis and molecular analysis

We harvested all plates three months after inoculation. We discarded contaminated plates and plates in which the fungal network did not cross into the central compartment. We removed roots from the plates and dried them in paper bags and extracted extraradical hyphae from the MSR medium (Whiteside et al., 2019). We weighed the dried root and fungal material and subsampled the roots for fluorescent analysis (~7 mg) and DNA extraction (~20 mg). To compare relative phosphorus transfer, we measured the quantum‐dot‐apatite fluorescence in the focal root systems with a Bio‐Tek Synergy MX plate reader as described in Whiteside et al. (2019). As a second metric to confirm that quantum‐dot‐apatite was being transferred as a phosphorus source to host roots, we determined total root phosphorus concentration via acid digestion and spectrophotometry in focal roots, following van 't Padje, Oyarte Galvez, et al. (2020). To measure intraradical fungal colonisation in the whole‐plant greenhouse and the in‐vitro root organ culture experiment, and extraradical fungal abundance in the in‐vitro root organ culture experiment, we isolated fungal DNA using the DNeasy Plant Mini kit (Qiagen) and analysed the fungal abundance with real time qPCR as described in Whiteside et al., 2019, allowing us to obtain total copy numbers of intra‐ and extraradical colonisation. It also allowed us to distinguish between *R*. *irregularis* and *R*. *aggregatum* when grown in combination. In contrast A5 and B12 are too genetically similar to use qPCR to differentiate their abundances. In those cases, only total abundance was measured. We tested the amplification efficiencies for each strain using the probes and primers designed by Kiers et al. (2011) (Table S1 in Kiers et al. (2011), Figure S8). To translate copy number to fungal biomass, we harvested fungal material from each strain, subsampled into samples ranging from 0.5 to 5 mg and extracted the DNA, and quantified copy numbers of each fungal subsample with qPCR as described above. We correlated the copy numbers to fungal biomass using linear regressions (Figure S9).

### Statistical analyses

We performed all statistical analysis in R version 3.3.1. We tested all data for normality of the residuals with a Shapiro test and transformed data by taking the square root or the logarithm if necessary. We analysed the data using linear models, with the independent variable as the partner strain (A5, B12 or Agg). We tested the homogeneity of the variances with Levene's test and checked the distribution of the residuals by eye with a normal QQ plot. We produced ANOVA type II tables with the R package car (Fox et al., 2019). To assess the statistical differences between the groups, we used a Tukey HSD test as post‐hoc test. We calculated fungal biomass using the functions to correlate fungal copy number to fungal biomass (Figure S9). To calculate the network efficiency, we calculated the amount of quantum‐dot‐apatite per total focal root over the focal extraradical hyphae (sum of fungal biomass in focal compartment and focal fungal biomass in the central compartment). All data supporting this research are available at the online data repository Zenodo, https://doi.org/10.5281/zenodo.5715280.

## RESULTS

### Fungal colonisation

**FIGURE 2:**
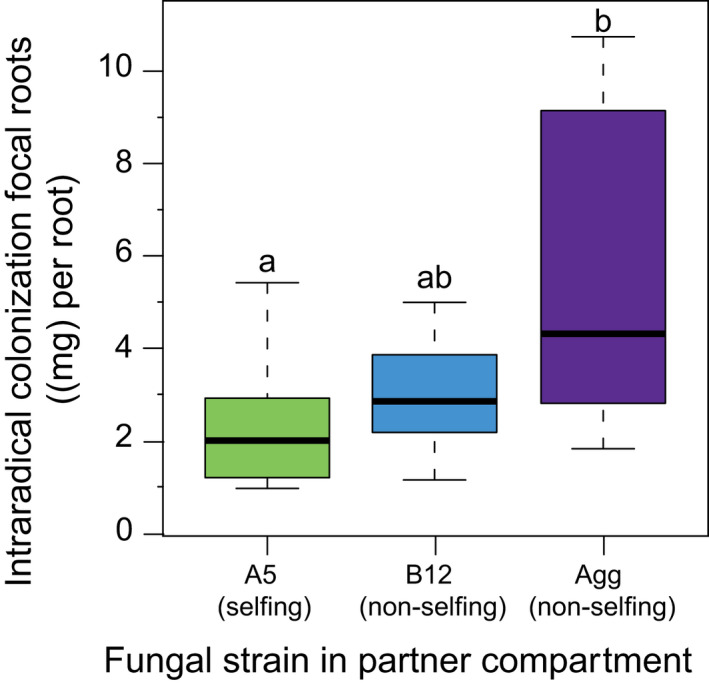
Boxplot of intraradical colonisation of focal roots (mg fungal biomass per root) in the whole‐plant greenhouse experiment. We found lower intraradical colonisation of focal roots when the fungal network was composed of less‐related, non‐selfing fungal strains. Box‐plots with different letters indicate significant difference (*p* < 0.05), top and bottom of the box indicate the first and third quartile, and the whiskers indicate the minimum and maximum values. *n*_A5_ = 8, *n*_B12_ = 9, *n*_Agg_ = 9

First, we analysed how level of fungal relatedness affected plant growth and fungal colonisation in the whole‐plant experiment. We found that intraradical colonisation of the focal plant root by A5 was significantly higher when grown with Agg. Specifically, intraradical colonisation of the focal root was increased by 34% when the partner plant was inoculated with the non‐selfing partner strain B12, and by 59% when the partner plant was inoculated with Agg (One‐way ANOVA: F_2,22_ = 4.7437, *p* = 0.0194) (Figure 2). Likewise, intraradical colonisation in partner plant roots was affected by relatedness treatment, with highest colonisation level when inoculated with Agg (One‐way ANOVA: F_2,21_ = 7.603, *p* = 0.003) (Figure S1).

**FIGURE 3:**
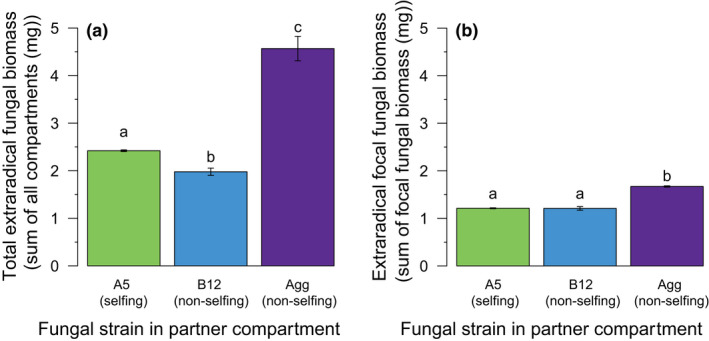
Barplots of total extraradical fungal abundance, focal extraradical fungal abundance. (a) The total extraradical fungal abundance (sum of all three compartments) is significantly influenced by the fungal strain in the partner compartment, with higher fungal abundance when the network is formed by A5‐Agg. (b) The amount of focal extraradical hyphae (sum of focal hyphae in focal and central compartment) is higher when the partner root is inoculated with Agg. Bars with different letters indicate significant difference (*p* < 0.05). *n*_A5_ = 12, *n*_B12_ = 11, *n*_Agg_ = 2

In the in‐vitro experiment, we quantified both extraradical and intraradical fungal abundance. We found a similar trend as in the whole‐plant experiment: total extraradical biomass (sum of all compartments) was roughly two times higher when the partner plant was inoculated with Agg compared to A5, and the lowest level of extraradical fungal with B12 (one‐way ANOVA: F_2,22_ = 18.236, *p* < 0.001) (Figure 3a). In particular, the extraradical biomass from focal hyphae (sum focal hyphae in focal and central compartment) was affected by the fungus in the partner compartment (ANOVA, F_2, 37_ = 166.46, *p* < 0.001): when the partner root was inoculated with Agg, the focal hyphae in the focal and central compartment formed 25% more extraradical fungal biomass (Figure 3b).

We then tested for intraradical fungal colonisation of the focal root in the in‐vitro experiment and found this did not differ statistically among the treatments (one‐way ANOVA: F_2,38_ = 2.023, *p* = 0.146) (Figure S2a). However, total intraradical colonisation of partner roots was significantly affected by the partner strain (one‐way ANOVA: F_2,38_ = 5.928, *p* = 0.006) (Figure S2b). As with the whole root experiment (Figure 2), roots inoculated with Agg had higher amounts of intraradical fungal biomass. Partner roots inoculated with Agg were also colonised by fungal strain A5 (0.804 ± 0.01 mg per root). Since A5 and Agg cannot fuse, this indicates that the fungus of the focal root crossed the central compartment and into the partner root compartment.

### Fungal network architecture

**FIGURE 4:**
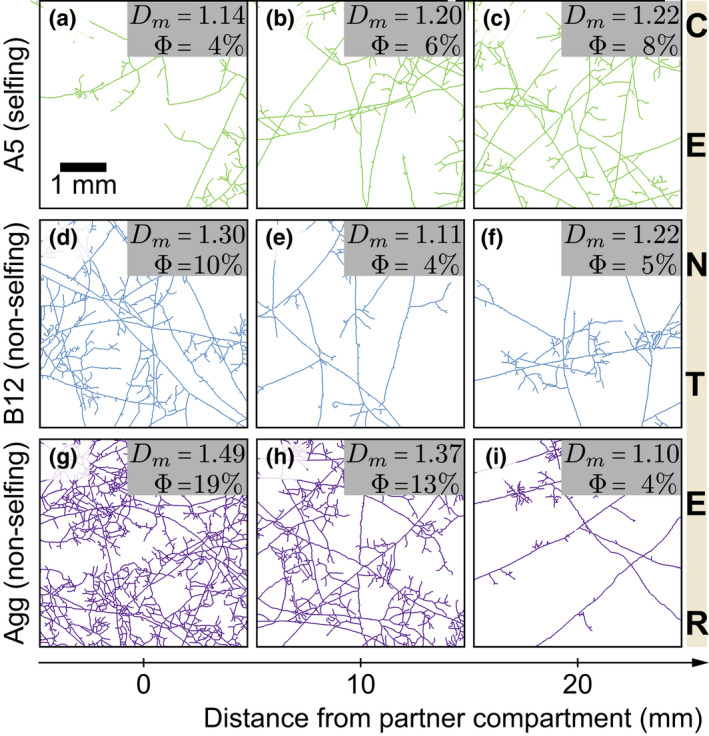
Physical architecture of extraradical network. Extraradical mycelium in the fungus‐only compartment is plotted from the partner compartment barrier (left) (a, d, g) to the centre (right) (c, f, i), for the three partner fungal strains. (a–c) A5 grows a denser and more complex network towards the centre of the central compartment, increasing the surface area and the *D_m_*. (d–f) B12 decreases in density and complexity towards the centre of the central compartment. (g–i) Agg shows the highest density and complexity near the partner compartment and the least towards the centre of the central compartment

We then studied the overall network architecture when the fungal strains were grown together. We found that the network architecture qualitatively differed with varying levels of fungal relatedness. Measuring from the focal root compartment toward the central fungus‐only compartment, we found that a network composed of only A5 increased in complexity from *D_m_* ~1.1 to *D_m_* ~1.2 moving toward the centre of the central compartment. This was accompanied by an increase in surface area covered from 4% to 8% (Figure 4a–c). However, when the partner root was inoculated with the non‐selfing partner strain B12 (Figure 4d–f) or Agg (Figure 4g–i), the complexity and density of the network showed the opposite pattern, with both strains decreasing towards the centre of the central compartment. We also grew the strains without a partner and found that when A5 was grown singly, there was no increase in surface area or complexity in the network as it grew away from the root. B12 also showed no change in complexity or density, while Agg showed high density and complexity closer to the root compartment (Figure S6).

### Nutrient transfer and host plant benefit

**FIGURE 5:**
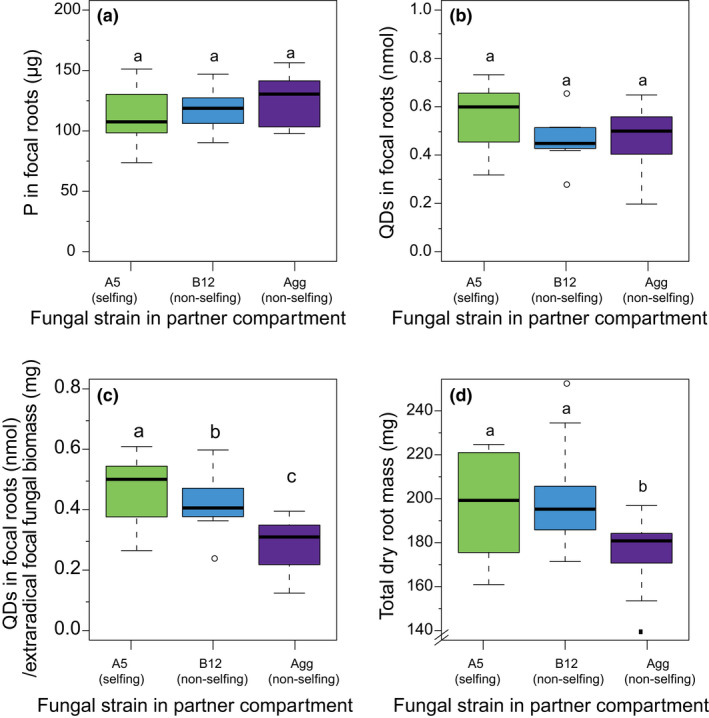
Box‐plots of the total phosphorus (P) as measured by acid digestion and total amount of quantum‐dot‐apatite (QD) in focal roots, fungal network efficiency and dry root weight of root organ cultures. (a) The total amount of phosphorus in focal roots was not significantly affected by the fungal strain in the partner compartment. *n*_A5_ = 12, *n*_B12_ = 8, *n*_Agg_ = 11. (b) The total amount of quantum‐dot‐apatite in the focal roots was not significantly affected by the fungal strain in the partner compartment. *n*_A5_ = 12, *n*_B12_ = 8, *n*_Agg_ = 12. (c) Quantum‐dot‐apatite transfer to the focal roots through focal hyphae was less efficient when associated with a less‐related fungal network. *n*_A5_ = 12, *n*_B12_ = 8, *n*_Agg_ = 12. (d) Total root mass (focal + partner root) was lower when the fungal network was less related. *n*_A5_ = 12, *n*_B12_ = 12, *n*_Agg_ = 17. Box‐plots with different letters indicate significant difference (*p* < 0.05), top and bottom of the box indicate the first and third quartile, and the whiskers indicate the minimum and maximum values

We next measured total phosphorus per root by both acid digestion and quantum‐dot‐apatite florescence. Neither total phosphorus in focal roots by acid digestion (one‐way ANOVA: F_2,28_ = 0.655, *p* = 0.537) (Figure 5a, Figure S6) nor total quantum‐dot‐apatite in focal roots (one‐way ANOVA: F_2,29_ = 1.698, *p* = 0.201) (Figure 5b) were significantly affected by relatedness treatment. However, we found that partner roots contained more phosphorus as measured by acid digestion when inoculated with A5 (Figure S6a). We then determined phosphorus transfer efficiency by measuring the amount of quantum‐dot‐apatite transferred to the focal host root per mg of extraradical focal fungal network and found that network relatedness had a significant effect on transfer efficiency (one‐way ANOVA: F_2,29_ = 9.444, *p *< 0.001) (Figure 5c). Specifically, A5‐A5 networks transferred on average of 10% more quantum‐dot‐apatite per mg of network than A5‐B12 networks, and 61% more than A5‐Agg networks. This result was confirmed by acid digestion measurements: total P per mg of extraradical fungal network in the focal compartment showed the highest fungal efficiency when the partner root was inoculated with A5 (Figure S7).

Lastly, we tested for effect of relatedness on plant biomass in both whole‐plant and in‐vitro experiments. In the whole‐plant experiment, we found no significant difference among total plant biomass across relatedness treatments in the time frame of our experiment (Figure S4). However, we did see a significant effect on the total root biomass accumulated in the in‐vitro experiment. Total root biomass of in‐vitro roots decreased when roots were connected with non‐selfing fungal strains (one‐way ANOVA: F_2,38_ = 5.396, *p* = 0.009). Specifically, total root biomass was 1.5% lower when the partner root was inoculated with A5‐B12 and 11% lower when the partner root was inoculated with A5‐Agg (Figure 5d).

## DISCUSSION

Our aim was to study the effects of varying fungal relatedness on nutrient transfer and network formation between host roots using both whole‐plants and in‐vitro root cultures. Using a whole‐plant system, we found that increased intraradical colonisation of the focal plant was associated with a partner plant inoculated with a non‐selfing, less‐related fungal strain (Figure 2). While this difference did not significantly affect overall plant biomass (Figure S4), it suggests that fungal competition underground may promote fungal colonisation, which could either be a carbon drain, or benefit to the host plant because of increased nutrient exchange (Campos et al., 2018; Grace et al., 2009). Our finding is in line with past work showing an increase in intraradical fungal abundance when a plant is inoculated simultaneously with several mycorrhizal fungal species (Jin et al., 2013).

Because accurately quantifying extraradical hyphal abundance in soil‐based systems is notoriously difficult (Fortin et al., 2002), we further tested this idea using a three‐compartment in vitro setup in which we could analyse the architecture of the extraradical network and harvest it in its entirety. Here, we also found that networks of non‐selfing fungal networks were associated with increases in extraradical fungal growth compared to A5‐A5 networks (Figure 3a,b). We did not find a statistically significant effect of relatedness on intraradical colonisation of the focal roots of the in‐vitro system (Figure S2a), but partner roots inoculated with Agg showed higher intraradical colonisation (Figure S2b). Similar results have been found in competition assays using both different species of arbuscular mycorrhizal fungi (Engelmoer et al., 2014) and different species of ectomycorrhizal fungi (Hortal et al., 2016). In these cases, it was suggested that allocation to growth in the soil could help maintain a competitive edge of fungi.

More generally, theory predicts that low genetic relatedness among parasites in hosts can increase competition and favour faster growth (Frank, 1996b; West, Kiers, et al., 2002). We found that inoculation of roots with different species, increased the competition between the arbuscular mycorrhizal fungi, which induced more extraradical growth (Figure 3a,b), especially dense near the partner root (Figure 4d–i). In contrast, A5‐A5 networks faced less competition for space and resources because there was the potential for fusion and resource sharing (Barreto de Novais et al., 2013; Croll et al., 2009; Giovannetti et al., 2001; Sbrana et al., 2011). Work is now needed to precisely quantify nutrient flows in fused versus non‐fused networks to understand how fusion affects nutrient transfer efficiency.

An open question is whether more extraradical growth changes the efficiency of nutrient transfer in fungal networks. We studied network efficiency by quantifying the total phosphorus in the roots by both acid digestion and the transfer of quantum‐dot‐apatite from the fungal network into the host roots. We added quantum‐dot‐apatite as a phosphorus source to the partner root compartment and determined how much was transferred from the fungal network into the focal root. This approached allowed us to compare cumulative patterns of phosphorus transfer from the network to the host root using visual florescence in the roots (van 't Padje, Oyarte Galvez, et al., 2020; van 't Padje, Werner 2020; van 't Padje et al., 2021; Whiteside et al., 2019). We found that roots contained more phosphorus (Figure S7), and that more quantum‐dot‐apatite was transferred to focal roots per mg focal extraradical fungal biomass when the roots were colonised by A5‐A5 networks (Figure 5c). Again, this increased efficiency is likely the result fusion in the central compartment of the selfing treatment, as found previously (Croll et al., 2009; Giovannetti & Sbrana, 2001). By fusing, fungi can tap into resources of already existing mycorrhizal fungal networks, increasing nutrient flow (Giovannetti et al., 2015; Novais et al., 2017; Pepe et al., 2016; Sbrana et al., 2011), and creating large, looping networks (Giovannetti et al., 2004), allowing for more nutrient transport across the network per unit fungus (Figure 5c). While only a qualitative comparison, we could visually document differences in growth strategies of the fungal network by extracting descriptive architecture data. We found that A5‐A5 networks formed denser networks in the central fungal compartment (Figure 4d–i). This increased density in the central compartment could be the result of increased fusion, as we did not see this increase when A5 was grown singly (Figure S5a–c). However, more work using precise time series is needed to confirm this hypothesis.

While significantly lower, we did find that there was still transfer of nutrients from the partner root to the focal root in non‐selfing networks, even in the absence of fusion. As confirmed by qPCR in the A5‐Agg treatment, this transfer is likely explained by the A5 strain from the focal root crossing the fungal compartment and colonising the partner root compartment. By crossing two physical barriers, A5 was able to form a continuous network between the two roots, facilitating movement of phosphorus between root compartments. Past work has confirmed that our plastic barriers prevent the passive diffusion of the quantum‐dot‐apatite across the plate (Whiteside et al., 2019). Therefore, any movement of tagged nutrients into the fungus‐only and focal root compartments is via the fungal network.

We found that decreased nutrient transfer efficiency of less‐related networks (Figure 5c; Figure S7) was translated into a growth cost for in‐vitro host roots, with lower total biomass of roots when inoculated with non‐selfing strains (Figure 5d). Taken together, this suggests that competition among fungi may drive an increase in fungal size, but not in phosphorus transfer benefits to the host. This result is in agreement with past work on these fungal strains suggesting that decreasing genetic relatedness within a single host root can decrease plant growth (Roger et al., 2013). It also agrees with work showing that plant productivity does not increase with the addition of more fungal species (Boyer et al., 2015; Jin et al., 2013; Lin et al., 2015; Van der Heijden et al., 2006). More fungal species can, depending on the specific plant‐fungal combinations, even decrease plant size (Jansa et al., 2008; Long et al., 2010).

More generally, our data suggest that decreased genetic relatedness in fungal networks can drive changes in the overall effectiveness of the symbioses. Being associated with a single genotype may lead to higher nutrient transfer efficiency because of decreased competition, and higher fusion. However, this idea needs to be tested more broadly using multiple strains for each level of relatedness, and in more heterogenous environments. This is important because, as the complexity of the environment increases, such that different strains are better able to acquire different or complementary resources, the benefits of interacting with a network of non‐relatives may likewise increase (Koide, 2000). In our case, the closely related – but not selfing—strains B12 and Agg potentially face very strong competition with A5, because they are not filling different ecological niches in our homogeneous, sterile cultures. In contrast, an A5‐A5 network faced no fungal competition because it was able to fuse and share resources with itself, across both plant roots. The ideal experimental design is one where relatedness is manipulated independent of diversity, as in biodiversity experiments (Wright et al., 2021), such that genetic relatedness is not confounded with function. Therefore, future work should focus on fungal strains with greater functional diversity, in more complex environments, to test whether the benefits for the host of accessing unique pools of resources outweigh the costs of interacting with competing fungal communities. These types of experiments are key in the testing of the costs and benefits of variation in symbiont relatedness.

## AUTHOR CONTRIBUTIONS

AP was responsible for the statistical analysis, writing and figure design, MK, VC, CB and NH designed the experiments and performed the experimental procedures. LOG and TS were responsible for image analysis, writing and figure design. IS provided the fungal strains and was involved in the experimental design and writing. ETK was involved in experimental design and writing.

### PEER REVIEW

The peer review history for this article is available at https://publons.com/publon/10.1111/ele.13947.

## Supporting information

Fig S1‐S9

## Data Availability

The authors confirm that the data supporting the results are deposited in the public repository Zenodo. https://doi.org/10.5281/zenodo.5715280.
