# Supplementary material for: Decreasing relatedness among mycorrhizal fungi in a shared plant network increases fungal network size but not plant benefit
Source: Ecol Lett. 2021 Dec 31;25(2):509–20. doi: 10.1111/ele.13947 (PMC9305232; doi:10.1111/ele.13947)
Supplement: Supplementary file 1 — Fig S1‐S9 [file ELE-25-509-s001.docx]

**Supporting information**

**Quantum-dot-apatite uptake**

Arbuscular mycorrhizal fungi are known for their ability to take up phosphorus, and exchange it with carbon from host roots (Jiang *et al.* 2017; Keymer *et al.* 2017; Luginbuehl *et al.* 2017). While the most well characterized pathway for phosphorus uptake is via high affinity orthophosphate transporters (Bun-Ya *et al.* 1991; Harrison & Buuren 1995; Maldonado-Mendoza *et al.* 2007) fungi have other means of nutrient uptake, most notably endocytic pathways. Clathrin-mediated endocytosis has been shown in the yeast *Candida albicans*, with invagination cells reaching diameters of 100 nm (Epp *et al.* 2013). Similar endocytosis processes have been demonstrated in the budding yeast *Saccharomyces cerevisiae* (Lu *et al.* 2016). Gustafsson *et al.* (2015) showed that yeast cells take up quantum-dot tagged glutathione using ADP1- encoded transporters. Likewise, endocytosis has been demonstrated to be important in the hyphae of filamentous fungi (Fischer-Parton *et al.* 2000; Read & Kalkman 2003). Uptake is first likely happening via enhanced dissolution of the apatite by mycorrhizal fungi (Powell & Daniel 1978; Alloush & Clark 2001; Pel et al. 2018), a process that will reduce the size of the tagged apatite crystals and facilitate endocytosis.

**Quantum-dot-apatite flow through hyphae**

Once inside the fungi, quantum-dot-apatite has been shown to move along fungal hyphae (van ’t Padje *et al.* 2020). Using synchronous bright field and fluorescence imaging, van ’t Padje *et al*. (2020) has shown how the hypha becomes full when QD-apatite is added to the medium - this can be seen changing the cytoplasmic flow. This is compared to the faster and unimpeded flow nutrients in hyphae lacking QD-apatite (supplementary video S2 in van ’t Padje *et al*. (2020). In the videos, van ’t Padje *et al*. (2020) use transmitted white light in order to observe the fungal hypha, as well as UV light through the objective lens for the excitation of the quantum-dot present in the P-apatite solution.

**Quantum-dot-apatite is used to build biological tissue**

There is evidence from two whole plant studies suggesting that phosphorus attached to quantum-dot-apatite is used to build biological plant tissue (Whiteside *et al.* 2019; van ’t Padje *et al.* 2021). Whiteside *et al.* (2019) tested this idea by inoculating *Medicago truncatula* with mycorrhizal fungi and fertilized them with apatite or quantum-dot tagged apatite as the only phosphorus source. If the phosphorus from the quantum-dot-apatite was not available to the plant, or if there was detoxification of nanoparticles, it would be expected that the plants grown on quantum-dot-apatite would show reduced growth compared to plants growing on apatite as phosphorus source. Instead, Whiteside *et al.* (2019) found no significant difference in plant growth between the plants growing on apatite and quantum-dot-apatite indicating that the quantum-dot-apatite was successfully used as a phosphorus source.

Work by van ’t Padje *et al.* (2021) also confirmed that the quantum-dot-apatite was transported to the growing leaves of plants, by measuring florescent profiles of the shoots of plants growing on quantum-dot-apatite. The shoots of plants growing on quantum-dot-apatite contained on average more quantum-dot-apatite than roots (van ’t Padje *et al.* 2021), indicating that the quantum-dots are transported to the growing tissue of the plants. Older work has also shown that quantum-dot tagged nutrients are transported to the mesophyll and even to the chloroplasts cells (Whiteside *et al.* 2009). Likewise, a higher percentage of phosphorus in host roots will originate from quantum-dot-apatite under low phosphorus conditions, suggesting quantum-dot-apatite is used as a phosphorus pool (van ’t Padje *et al.* (2020).

In addition, acid digestion P-measurement methods have been used to further confirm that quantum-dot**s** give an accurate measurement for phosphorus transferred to roots. van ’t Padje *et al.* (2020) found that approximately 6% of total phosphorus in *in-vitro* roots originated from quantum-dots. Our measurements of total phosphorus, also measured by acid digestion, in roots find roughly the same amount (Fig 5, S6).

**Quantum-dots lacking phosphorus are not taken up by fungi**

Whiteside *et al.* (2019) demonstrated that quantum-dots - in the absence of apatite – will not be taken up by arbuscular mycorrhizal fungi. Specifically, when Whiteside *et al.* (2019) exposed fungal hyphae to unconjugated carboxyl terminated quantum-dots, no uptake or translocation of quantum-dots to the root (after 60 days of exposure was observed: the amount of quantum-dot**-**apatite was lower than the detection limit (< 0.000001 nmol quantum-dot mg^-1^ plant tissue). Likewise, fungi exposed to bare metal quantum-dot cores (i.e “naked quantum dots), values in root tissue were also below detection levels (< 0.000001 nmol quantum-dot mg^-1^ plant tissue) (Whiteside *et al.* 2019). This provides evidence that nutrient conjugation is the key cue for the uptake and transfer across biological tissue, suggesting a shell of mineral apatite remains bound the quantum-dot core.

**Direct quantum-dot-apatite uptake**

Whiteside et al. (2019) demonstrated the direct uptake of quantum-dot-apatite into host roots using a split-root whole-plant design in which half the roots were inoculated with mycorrhizal fungi, and half were not. In this experiment, quantum-dot-apatite of two different colors were added to each root half (and also switched the colors for a color control). The data suggest that tagged quantum-dots can be taken up by roots in the absence of mycorrhizae, but that this uptake is significantly lower (Whiteside et al. 2019). Past work in other labs has shown that receptor-mediated endocytosis in plant roots can be important in the direct uptake of quantum-dots (Etxeberria *et al.* 2006; Šamaj 2012). Cell wall pores have been measured previously by (McCann *et al.* 1990), they found that the diameter varied between 5-20 nm, large enough to allow passage of partly dissolved quantum-dot-apatite. Once inside the plant cells, the partly dissolute quantum-dots can cross the cells simplistically via cell wall pores. Transport of nanoparticles can also occur apoplastically and via the vascular system of the plants (reviewed in Schwab et al. 2016).

**Open Questions**

While studies are accumulating data and video imaging provide evidence of quantum-dot-apatite uptake and translocation by mycorrhizal fungi to the host plant, a harder question is quantifying the rate at which phosphorus is dissociated from the quantum-dot core across different biological tissue. Various options using anisotropy approaches, which involve measuring the rotational speed of partly dissolved versus fully intact quantum-dot-apatite will likely be used in the future to tackle this question, as florescence particles are more widely used to track nutrients in ecological studies.

**References**

**Bun-Ya, M., Nishimura, M., Harashima, S. & Oshima, Y.** (1991). The PHO84 gene of Saccharomyces cerevisiae encodes an inorganic phosphate transporter. *Mol. Cell. Biol.*, 11, 3229–3238, doi: 10.1128/MCB.11.6.3229.

**Epp, E., Nazarova, E., Regan, H., Douglas, L.M., Konopka, J.B., Vogel, J., *et al.*** (2013). Clathrin- and arp2/3-independent endocytosis in the fungal pathogen Candida albicans. *MBio*, 4, e00476-13, doi: 10.1128/mBio.00476-13.

**Etxeberria, E., Gonzalez, P., Baroja-Fernández, E. & Romero, J.P.** (2006). Fluid Phase Endocytic Uptake of Artificial Nano-Spheres and Fluorescent Quantum Dots by Sycamore Cultured Cells. *Plant Signal. Behav.*, 1, 196–200, doi: 10.4161/psb.1.4.3142.

**Fischer-Parton, S., Parton, R.M., Hickey, P.C., Dijksterhuis, J., Atkinson, H.A. & Read, N.D.** (2000). Confocal microscopy of FM4-64 as a tool for analysing endocytosis and vesicle trafficking in living fungal hyphae. *J. Microsc.*, 198, 246–259, doi: 10.1046/j.1365-2818.2000.00708.x.

**Gustafsson, F.S., Whiteside, M.D., Jiranek, V. & Durall, D.M.** (2015). Development and use of a quantum dot probe to track multiple yeast strains in mixed culture. *Sci. Rep.*, 4, 6971, doi: 10.1038/srep06971.

**Harrison, M.J. & Buuren, M.L. Van**. (1995). A phosphate transporter from the mycorrhizal fungus Glomus versiforme. *Nature*, 378, 19–22, .

**Jiang, Y., Wang, W., Xie, Q., Liu, N., Liu, L., Wang, D., *et al.*** (2017). Plants transfer lipids to sustain colonization by mutualistic mycorrhizal and parasitic fungi. *Science.*, 356, 1172–1173, doi: 10.1126/science.aam9970.

**Keymer, A., Pimprikar, P., Wewer, V., Huber, C., Brands, M., Bucerius, S.L., *et al.*** (2017). Lipid transfer from plants to arbuscular mycorrhiza fungi. *Elife*, 6, 1–33, doi: 10.7554/eLife.29107.

**Lu, R., Drubin, D.G. & Sun, Y.** (2016). Clathrin-mediated endocytosis in budding yeast at a glance. *J. Cell Sci.*, 129, 1531–1536, doi: 10.1242/jcs.182303.

**Luginbuehl, L.H., Menard, G.N., Kurup, S., Van Erp, H., Radhakrishnan, G. V., Breakspear, A., *et al.*** (2017). Fatty acids in arbuscular mycorrhizal fungi are synthesized by the host plant. *Science.*, 356, 1175–1178, doi: 10.1126/science.aan0081.

**Maldonado-Mendoza, I.E., Dewbre, G.R. & Harrison, M.J.** (2007). A Phosphate Transporter Gene from the Extra-Radical Mycelium of an Arbuscular Mycorrhizal Fungus Glomus intraradices Is Regulated in Response to Phosphate in the Environment . *Mol. Plant-Microbe Interact.*, 14, 1140–1148, doi: 10.1094/mpmi.2001.14.10.1140.

**McCann, M.C., Wells, B. & Roberts, K.** (1990). Direct visualization of cross-links in the primary plant cell wall. *J. Cell Sci.*, 96, 323–334, .

**Read, N.D. & Kalkman, E.R.** (2003). Does endocytosis occur in fungal hyphae? *Fungal Genet. Biol.*, 39, 199–203, doi: 10.1016/S1087-1845(03)00045-8.

**Šamaj, J.** (2012). *Endocytosis in plants*. Springer, Berlin & Heidelberg, Germany.

**Schwab, F., Zhai, G., Kern, M., Turner, A., Schnoor, J.L. & Wiesner, M.R.** (2016). Barriers, pathways and processes for uptake, translocation and accumulation of nanomaterials in plants - Critical review. *Nanotoxicology*, 10, 257–278, doi: 10.3109/17435390.2015.1048326.

**van ’t Padje, A., Bonfante, P., Ciampi, L.T. & Kiers, E.T.** (2021). Quantifying Nutrient Trade in the Arbuscular Mycorrhizal Symbiosis Under Extreme Weather Events Using Quantum-Dot Tagged Phosphorus. *Front. Ecol. Evol.*, 9, 1–13, doi: 10.3389/fevo.2021.613119.

**van ’t Padje, A., Oyarte Galvez, L., Klein, M., Hink, M.A., Postma, M., Shimizu, T., *et al.*** (2020). Temporal tracking of quantum-dot apatite across in vitro mycorrhizal networks shows how host demand can influence fungal nutrient transfer strategies. *ISME J.*, 15, 435–449, doi: 10.1038/s41396-020-00786-w.

**Whiteside, M.D., Treseder, K.K. & Atsatt, P.R.** (2009). The brighter side of soils: Quantum dots track organic nitrogen through fungi and plants. *Ecology*, 90, 100–108, doi: 10.1890/07-2115.1.

**Whiteside, M.D., Werner, G.D.A.A., Caldas, V.E.A., van ’t Padje, A., Dupin, S.E., Elbers, B., *et al.*** (2019). Mycorrhizal Fungi Respond to Resource Inequality by Moving Phosphorus from Rich to Poor Patches across Networks. *Curr. Biol.*, 29, 1–8, doi: 10.1016/j.cub.2019.04.061.

**Supporting Figures**


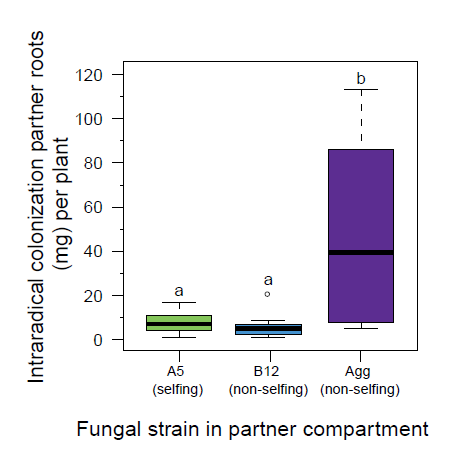


**Fig. S1. Boxplot of intraradical colonization of the partner roots (mg/plant) of the whole plant greenhouse experiment, per partner fungus**. We found a significant effect of the fungal partner strain on the colonization of partner plants. We found the highest colonization of partner plants which were inoculated by Agg (purple). Box-plots with different letters indicate significant difference (p < 0.05), top and bottom of the box indicate the first and third quartile, and the whiskers indicate the minimum and maximum values. *n*_A5_ = 8, *n*_B12_=7, *n*_Agg_=9.


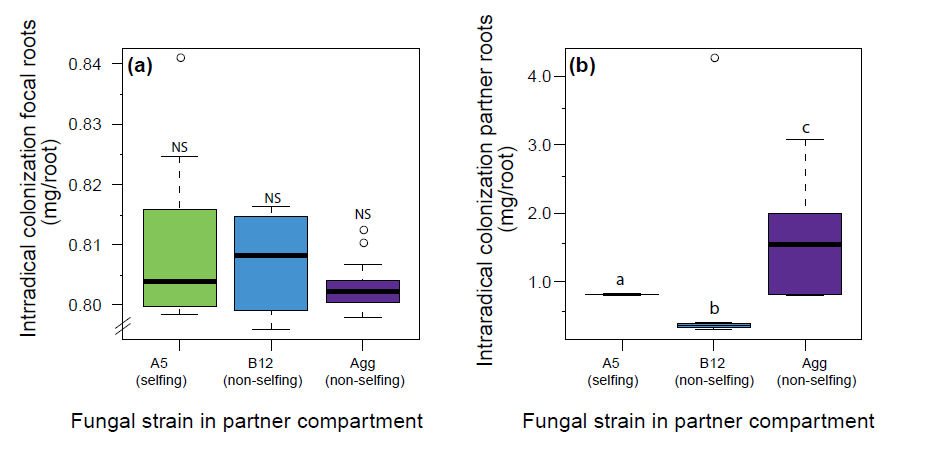


**Fig. S2. Boxplots of intraradical colonization *in vitro* root organ cultures (mg/root)**.

**(a)** Intraradical colonization of the focal root was not significantly affected by the partner fungus. **(b)** Intraradical colonization of the partner root was significantly influenced by the partner fungus, with the highest colonization of partner roots inoculated with Agg. Box-plots with different letters indicate significant difference (p < 0.05), top and bottom of the box indicate the first and third quartile, and the whiskers indicate the minimum and maximum values. *n*_A5_=12, *n*_B12_=12, *n*_Agg_=17.


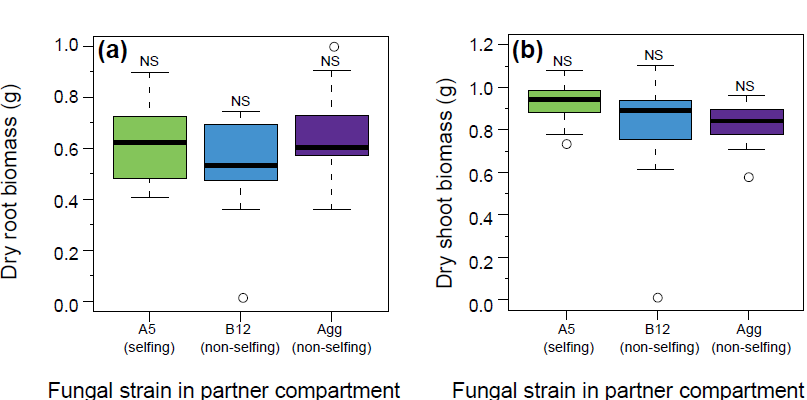


**Fig. S3. Boxplots of single plant biomass when grown with a single fungal strain.** **(a)** Root biomass was not significantly influenced by the fungal strain in the partner compartment (one-way ANOVA: F_2,40_=0.191, p=0.827). **(b)** We also found that the shoot biomass was not significantly influenced by the partner fungus (one-way ANOVA: F_2,40_=3.168, p=0.053). Box-plots with different letters indicate significant difference (p < 0.05), top and bottom of the box indicate the first and third quartile, and the whiskers indicate the minimum and maximum values. *n*_A5_=15, *n*_B12_=13, *n*_Agg_=15.


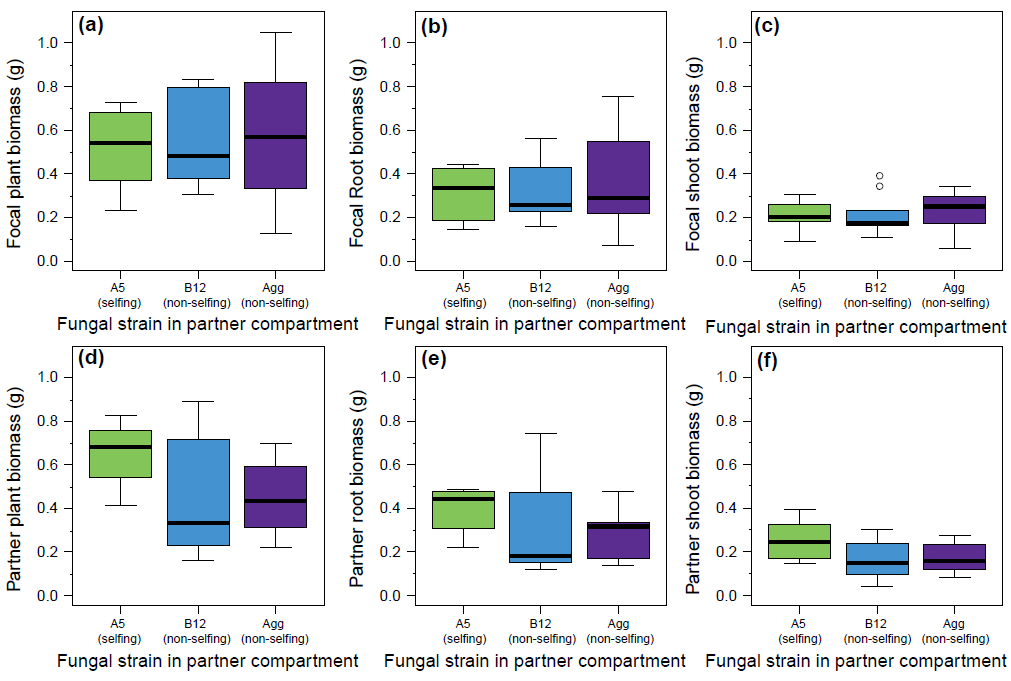


**Fig. S4. Boxplots of biomass focal and partner plants of the whole plant greenhouse experiment by partner fungus.** Growth of the focal plant was not significantly influenced by the partner strain (one-way ANOVA: **(a)** total biomass focal plant: F_2,23_=0.229, p=0.797, **(b)** root biomass focal plant: F_2,23_=0.401, p=0.674; **(c)** shoot biomass focal plant: F_2,23_=0.011, p=0.989), nor the partner plant (one-way ANOVA: **(d)** total biomass partner plant: F_2,23_=0.120, p=0.120, **(e)** root biomass partner plant: F_2,23_=1.523, p=0. 239; **(f)** shoot biomass partner plant: F_2,23_=3.034, p=0.067). Top and bottom of the box indicate the first and third quartile, and the whiskers indicate the minimum and maximum values. *n*_A5_=8, *n*_B12_=9, *n*_Agg_=9.


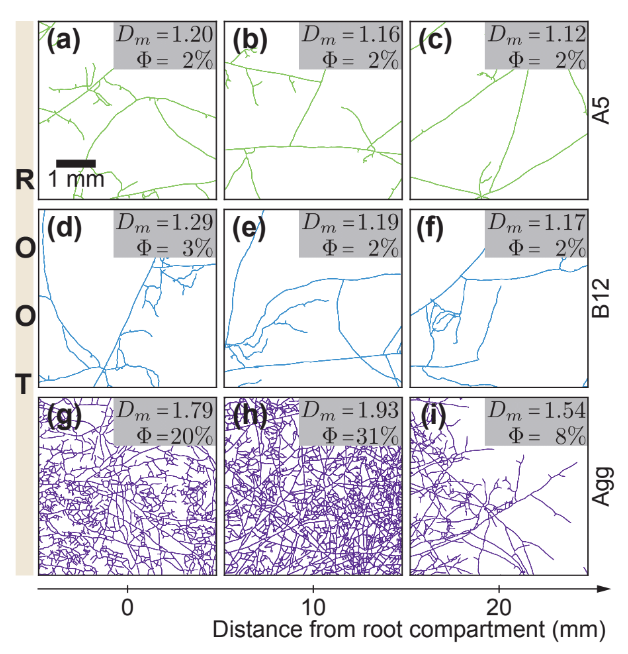


**Fig. S5. Physical architecture of extraradical fungal network of single fungal cultures.** The extraradical fungal network in the fungus-only compartment is plotted from the root compartment barrier (left) (a), (d), (g) to the fungal growth direction (right) (c), (f), (i).


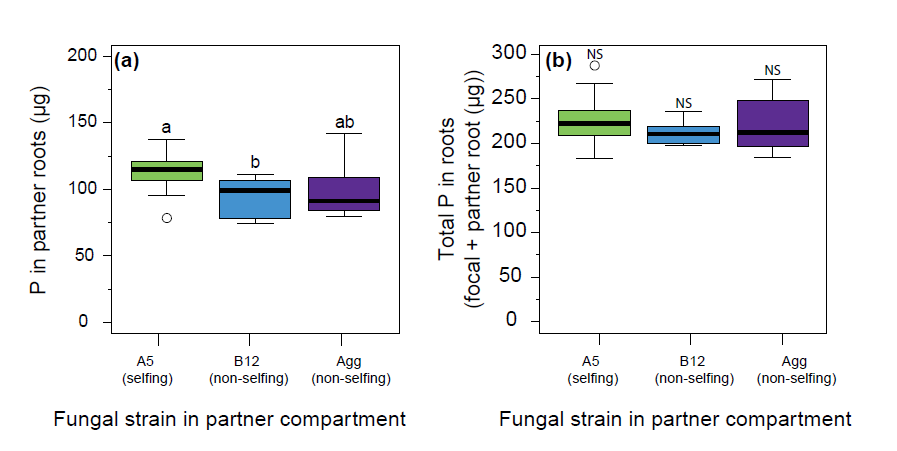


**Fig. S6. Phosphorus (P) in partner roots and total phosphorus in partner and focal roots in the *in-vitro* root experiment. (a)** Phosphorus in the partner roots was significantly affected by the fungus in the partner compartment (one-way ANOVA, F_2,28_=3.431, p=0.046). **(b)** Total phosphorus content of focal plus partner roots was not significantly affected by the fungus in the partner compartment (one-way ANOVA, F_2,28_=0.7132, p=0.499). Top and bottom of the box indicate the first and third quartile, and the whiskers indicate the minimum and maximum values. *n*_A5_=12, *n*_B12_=8, *n*_Agg_=11.


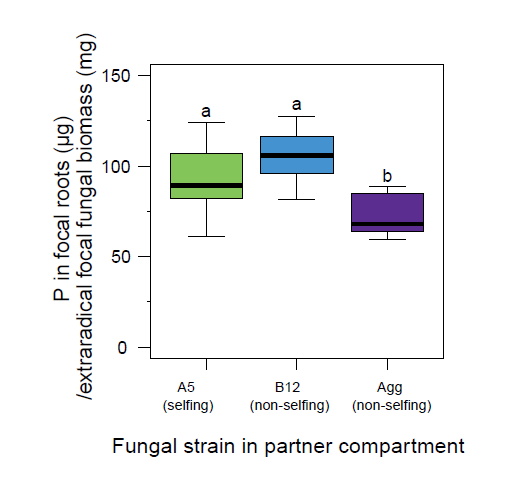


**Fig. S7. The amount of phosphorus (P) in the focal roots over the amount of focal extraradical biomass (sum of focal compartment and focal hyphae in central compartment) in the *in-vitro* root experiment.** Phosphorus per mg of extraradical hyphae in the focal roots higher was higher in the A5 and B12 treatment compared to when the partner roots were inoculated with Agg (one-way ANOVA, F_2,28_=11.167 p=0.0003). Top and bottom of the box indicate the first and third quartile, and the whiskers indicate the minimum and maximum values. *n*_A5_=12, *n*_B12_=8, *n*_Agg_=11.


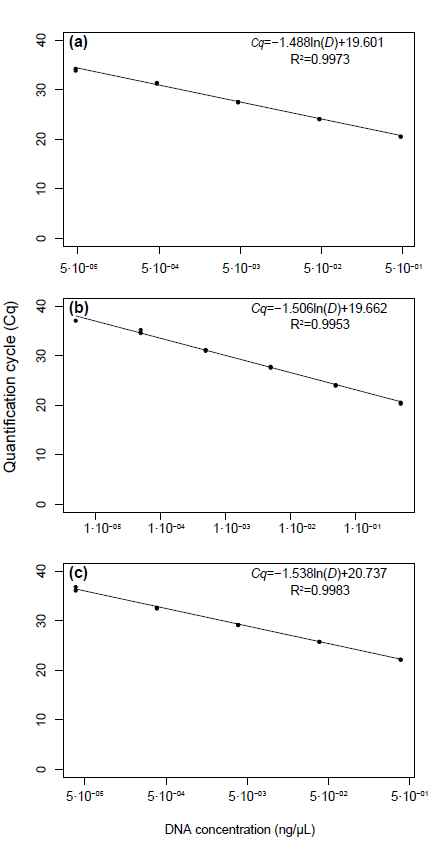


**Fig. S8. Amplification efficiency of the three fungal strains**. **(a)** The amplification efficiency (E= -1+10^-1/slope^) of *R. irregularis* strain A5 was 78.721%. **(b)** The amplification efficiency of *R. irregularis* strain B12 was 77.124%. **(c)** The amplification efficiency of *R. aggregatum* was 77.623%. The equations for the conversion of qPCR signal (*Cq* or quantification cycle) to fungal DNA concentration (*D*) are given for each fungal strain.

**
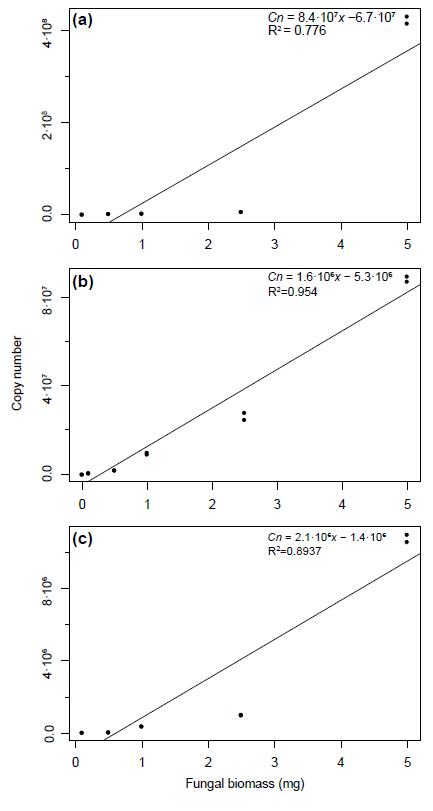
­Fig. S9. Calibration curves for the conversion of copy number of target genes to fungal biomass. (a)** *R. irregularis* strain A5. **(b)** *R. irregularis* strain B12. **(c)** *R. aggregatum*. The equation for the conversion from fungal copy number (Cn) to fungal biomass (x) are given for each fungal strain.
